# Immunohistochemical Expression of Baculoviral Inhibitor of Apoptotic Proteins Repeat-Containing Protein in Tumors of Salivary Gland Origin 

**DOI:** 10.30476/dentjods.2024.99323.2139

**Published:** 2025-06-01

**Authors:** Shima Torabi Ardekani, Hosein Mirhadi, Seyed Ali Ghaboos, Raziyeh Zare, Marzieh Khajeh

**Affiliations:** 1 Dept. of Oral and Maxillofacial Pathology, School of Dentistry, Shiraz University of Medical Sciences, Shiraz, Iran.; 2 Dept. of Endodontics, School of Dentistry, Shiraz University of Medical Sciences, Shiraz, Iran.; 3 Dept. of Orthodontics, School of Dentistry, Shiraz University of Medical Sciences, Shiraz, Iran.; 4 Student, School of Dentistry, Shiraz University of Medical Sciences, Shiraz, Iran.

**Keywords:** Salivary gland neoplasms, Adenoma, Pleomorphic, Carcinoma, Mucoepidermoid, Carcinoma, Adenoid cystic, Immunohistochemistry

## Abstract

**Background:**

Salivary gland tumors (SGTs) include benign and malignant tumors, such as pleomorphic adenoma (PA), mucoepidermoid carcinoma (MEC), and adenoid cystic carcinoma (ACC). Baculoviral inhibitors of apoptotic proteins (BIAPs) repeat-containing protein 6 (BIRC6), is an anti-apoptotic protein that plays an important role in cancers.

**Purpose:**

We aimed to evaluate the expression of BIRC6 in SGTs and its correlation with the clinicopathological features.

**Materials and Method:**

In this cross-sectional study, 56 SGT tissue samples, including 15 cases of MEC, 20 cases of ACC, and 21 cases of PA, as well as nine cases of normal salivary gland tissues, were investigated for BIRC6 expression by immunohistochemical analysis.

**Results:**

BIRC6 was found in 2.50%, 63%, 88%, and 63% of normal tissue, MEC, ACC, and PA, respectively. The mean total score of BIRC6 expression was 9.13; for ACC, MEC, PA, and normal tissue of the salivary gland were 10.25, 9.13, 9.00, and 3.00, respectively. The high expression of BIRC6 was detected in 29 cases of malignant and 15 cases of benign SGTs. The frequency of high or low expression was not different between the male and female patients (*p*= 0.833), patients aged below or above 65 years (*p*= 0.950), patients with different tumor sizes (*p*= 0.734), and metastasis status (*p*= 0.977).

**Conclusion:**

The higher expression intensity and percentage of BIRC6 in malignant and benign SGTs suggests it as a potential marker to be used in future targeted therapy for SGTs.

## Introduction

Salivary gland tumors (SGTs) are rare neoplasms of the head and neck (3-6%), comprising about 0.5-1% of all tumors [ 1 ], mainly present in the fifth or sixth decade of life [ 2 ] with an average annual age-adjusted incidence rate of 3.3:100,000 population for benign tumors and 0.8 for malignant tumors. The most common SGTs include pleomorphic adenoma (PA) (the most common benign tumor), mucoepidermoid carcinoma (MEC) and adenoid cystic carcinoma (ACC) (the most common malignant tumors), in addition to invasive tumors with a high rate of metastasis [ 3 ]. 

One of the main challenges in SGTs is the diagnosis, as most of the cases are asymptomatic or present with mild and chronic symptoms, such as painless swelling or facial pain. In the meantime, the histological heterogeneity makes the diagnosis more difficult and complex [ 4 ]. This is while the patients’ prognosis and treatment success depend on the tumor stage and invasion; therefore, on-time and early diagnosis are foremost important [ 5 ]. Accordingly, recent research has focused on different molecular biomarkers, which can help in the diagnosis and/or differentiation of SGT’s type by the use of immunohistochemistry (IHC) . 

Baculoviral inhibitor of apoptotic proteins (BIAPs) repeat-containing protein 6 (BIRC6), also known as apollon, is a large protein at 528 kDa and one of the eight members of inhibitor of apoptotic proteins (IAPs), which can inhibit caspase cascade and apoptosis [ 8 ]. BIRC6 is found abundantly in the brain, testis, bone marrow, lymphoid, and endocrine tissues [ 8 ] and plays a key role in the survival and patients’ prognosis as well as resistance/response to treatment in different types of cancers, such as lung cancers [ 9 ], ovarian cancers [ 10 ], colorectal cancers [ 11 ], and prostate cancers [ 12 ] and suggested as a novel biomarker for targeted anticancer therapy. 

Biomarkers play an important role in novel anticancer treatment strategies [ 13 ], and several biomarkers have been investigated and approved for diagnostic, prognostic, and treatment targets in SGTs [ 14 - 16 ]. However, a role for BIRC6 in salivary gland tumors has not been reported so far. Only in one study was the expression of BIRC6 evaluated in 49 patients with ACC, which reported zero overexpression of BIRC6 in the studied cases [ 17 ]. Therefore, in the present study, we aimed to evaluate the expression level and percentage of BIRC6 in three types of salivary gland tumors and the correlation of BIRC6 overexpression with the clinicopathological features. 

## Materials and Method

### Study design

The study protocol received approval from the Ethics Committee of Shiraz University of Medical Sciences (IR.SUMS.REC.1397.340), and necessary permissions were obtained from the hospital manager. Tissue samples with salivary gland tumors, preserved at Khalili Hospital in Shiraz, Iran, until autumn 2014, were collected. The blocks stained with Hematoxylin and Eosin (H&E) were assessed, and slides containing an adequate cellular sample (N=56) were included in the study. Additionally, nine samples of normal salivary gland tissues were chosen as controls. 

The samples underwent immunohistochemical staining using the Novocastra IHC Diluent system and Novolink Polymer Detection System. To achieve this, the samples were initially fixed in a formalin buffer and embedded in paraffin. Subsequently, 5 µm–thick slides were prepared, mounted, deparaffinized using Xylene, and washed in dehydrated alcohol and distilled water. Antigen retrieval was performed using a 0.09% sodium azide buffer with a pH of 9 for 20 minutes. The activity of intercellular peroxidase was inhibited by 10% H2O2. 

The primary antibody, Anti-BIRC6, was a rabbit polyclonal antibody (AB-19609) diluted with phosphate buffered saline (PBS) at a ratio of 1:3500 and maintained at room temperature for the assessment of the antigen-antibody reaction. Subsequently, the samples were stained using 3,3 diaminobenzidine (DAB), Liquid K3467 from Dako Corp., Denmark, serving as the chromogen. Harris’ hematoxylin was applied as a counterstain. Following staining, the slides were washed with tap water, dried, and then covered with a lamella. 

The brain tumor tissue samples served as a positive control for BIRC6, with brown cytoplasmic coloring considered indicative of positive BIRC6 presence. Evaluation of stained slides was conducted using a light microscope by two independent pathologists to minimize observer error. In instances of heterogeneous staining, sections with more pronounced staining were meticulously assessed. Randomly selected sections underwent cell counting, with 100 cells assessed at a magnification of 400×, and the percentage of cell staining was determined. 

**Figure 1:**
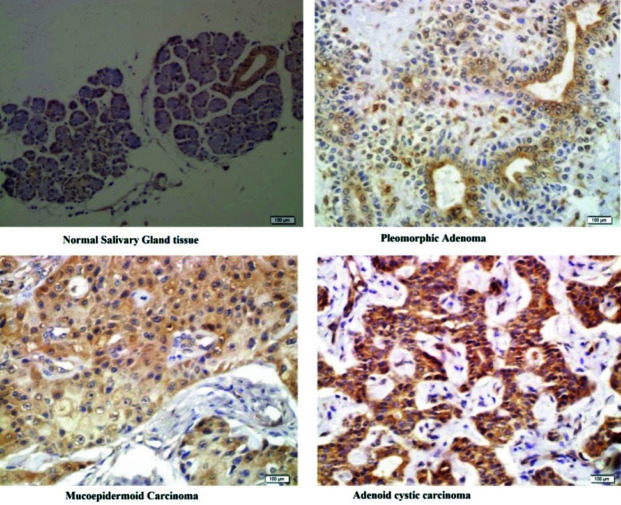
Cytoplasmic satiability of the four study groups evaluated in the present study

To gauge the staining degree of the cells, all slides were compared with the positive control exhibiting the same stainability. Figure 1 illustrates the cytoplasmic stainability across groups containing normal salivary gland tumors, PA, MEC, and ACC. In normal salivary gland tumors, ductal and myoepithelial cells tested positive for BIRC6, while mucous cells exhibited negative staining. Among the tumor groups, both epithelial and myoepithelial cells were positive for BIRC6. 

A semi-qualitative grading system was employed to assess the expression of BIRC6 in tumoral cells and normal salivary gland cells. For the expression level, grade 0 indicated 0-5%, grade 1 for 6-25%, grade 2 for 26-50%, grade 3 for 51-75%, and grade 4 for >76%. Regarding cell stainability, grade 0 denoted cells without staining, grade 1 for mild staining, grade 2 for moderate staining, and grade 3 for strong staining [ 18 - 19 ]. 

Total scores were calculated by multiplying the degree of marker expression by the degree of staining, resulting in scores of 0, 1, 2, 3, 4, 6, 8, 9, and 12. Scores exceeding 6 were considered indicative of high BIRC6 expression, while scores of 6 or below were categorized as low expression [ 19 ]. 

### Statistical analysis

The study findings were presented using Mean± Standard deviation (SD) for quantitative variables and frequency (percentage) for categorical variables. The normal distribution of variables was assessed using the One-sample Kolmogorov-Smirnov test, and Levene's test was employed to evaluate the equality of variances. Since these tests indicated normal distribution and confirmed equality of variances, continuous variables were compared using the Student t-test, while categorical variables were assessed using the Chi-square test. 

Statistical analysis was conducted using IBM SPSS Statistics for Windows version 21.0 (IBM Corp. 2012, Armonk, NY: IBM Corp.). A significance level of *p*< 0.05 was considered statistically significant. 

## Results

Of the 65 samples evaluated, nine were normal salivary gland tissues, and 56 were salivary gland tumor tissues, which included 15 cases of MEC, 20 cases of ACC, and 21 cases of PA. Of all tumor cells, 21 cases were in minor salivary glands (36.2%), 32 cases in major salivary glands (55.17%), and 12 cases were undetermined (20.68%). The mean age, sex distribution of patients, and the frequency of the involved salivary gland were compared among subgroups, as presented in Table 1. 

**Table 1:** The frequency of demographic variables in the study samples and type of the involved salivary gland in each subgroup

Variables	Categories	Normal tissue (N=9)	Pleomorphic adenoma (N=20)	Adenoid cystic carcinoma (N=17)	Mucoepidermoid carcinoma (N=14)
Age (years), mean±SD		40.5±12.36	35.40±16.96	52.94±15.30	53.57±19.44
Sex distribution, No. (%)	Female	5 (55.5)	12 (60)	9 (52.9)	9 (64.3)
	Male	4 (44.4)	8 (40)	8 (40.1)	5 (35.7)
Type of salivary gland	Major	28.6%	89.5%	31.2%	72.7%
	Minor	71.4%	10.5%	68.8%	27.3%

As demonstrated in Table 2, there was a significant difference among the four groups in the frequency of BIRC6 expression intensity and percentage (*p*< 0.001), and the frequency of expression of 51-75% and 76-100% were higher in ACC and MEC than other tumors and normal tissue (Figure 1). The mean expression percentage and the mean total scores of the groups, as well as the frequency of high/low expression of BIRC6 are shown in Table 2. The smallest mean total score was observed in normal tissues, but the pairwise comparison of mean scores among tumor cell tissues showed no significant difference between PA and MEC (*p*= 1.00), PA and ACC (*p*= 0.815), and MEC and ACC (*p*= 1.00). 

**Table 2:** Comparing the expression of baculoviral inhibitor repeat-containing protein 6 (BIRC6) in each subgroup

Variables	Categories	Normal tissue (N=9)	Mucoepidermoid carcinoma (N=14)	Adenoid cystic carcinoma (N=17)	Pleomorphic adenoma (N=20)	*p* value*
Expression intensity, No. (%)	Low	3 (33.3)	–	–	–	0.0008
	Moderate	2 (22.2)	6 (40)	3 (15)	6 (27.3)	
	High	4 (44.4)	9 (60)	17 (85)	6 (72.7)	
Expression percentage, No. (%)	0–5%	0	0	0	0	0.0001
	6–25%	6 (66.7)	0	0	0	
	26–50%	3 (33.3)	2 (10)	1 (6.7)	4 (19)	
	51–75%	0	5 (25)	6 (40)	7 (33.3)	
	76–100%	0	13 (65)	8 (53.5)	10 (47.6)	
Mean expression percentage		2.5%	63%	88%	63%	-
Mean total score, mean±SD		3±2	9.13±2.77	10.25±2.59	9±2.83	-
High expression of BIRC6, No. (%)		0	11 (73.3)	18 (90)	15 (71.4)	-
Low expression of BIRC6, No. (%)		9 (100)	4 (26.7)	2 (10)	6 (28.6)	-

*The results of Chi-square test, considered significant at *p*< 0.05

The comparison of the cytoplasmic expression frequency of the BIRC6 protein (categorized as high or low) with respect to demographic and clinical variables revealed no significant differences in the frequency of high or low BIRC6 expression between male and female patients (*p*= 0.833), patients aged below or above 65 years (*p*= 0.950), different tumor sizes (both *p*= 0.734), and metastasis status (*p*= 0.977), as shown in Table 3. 

**Table 3:** The difference in the frequency of high/low expression of baculoviral inhibitor repeat-containing protein 6 (BIRC6) based on the demographic and clinical variables

Variables	Number	High expression of BIRC6	Low expression of BIRC6	*p* value*
Male, No. (%)	24	16 (40)	7 (38.9)	0.8336
Female, No. (%)	36	24 (60)	11 (61.1)	
Age<65 years	12	8 (20)	3 (16.7)	0.9502
Age≥65 years	48	32 (80)	15 (83.3)	
Tumor sizes 1 and 2	19 (63.33)	16 (64)	3 (60)	0.7347
Tumor sizes 3 and 4	11 (36.67)	9 (36)	2 (40)	
Metastasis to lymph nodes	3 (10.34)	3 (12.5)	0	0.9778
Stages 1 and 2	19 (63.33)	16 (64)	3 (60)	0.7347
Stages 3 and 4	11 (36.67)	2 (40)	9 (36)	

*The results of the Chi-square test, considered significant at *p*< 0.05

## Discussion

The evaluation of 56 tissue samples with three types of SGTs, compared with nine normal salivary gland tissues in the present study, showed significantly higher expression percentage, mean expression score, and higher frequency of BIRC6 overexpression in samples with SGT compared with normal salivary gland tissues. These results indicate an important role for BIRC6 in SGTs. 

Apoptosis is considered a protective agent against uncontrolled cell proliferation in cancers, and apoptosis inhibition is considered one of the important triggers of cancers, which has suggested the use of apoptosis inhibitors as an anticancer treatment target [ 20 ]. BIRC6 is among the apoptosis inhibitors, and prior studies have investigated its role in various cancers, proposing that BIRC6 has the capability to degrade pro-apoptotic proteins [ 21 - 22 ] and deregulate extrinsic and intrinsic cell death pathways [ 23 - 24 ]. Identification of the significant role of BIRC6 in cancer progression and response to treatment has resulted in the suggestion of BIRC6 as a novel target for anticancer therapy in different cancers, such as prostate cancer [ 12 , 25 - 26 ]. However, its role in different types of SGTs has not been evaluated so far. Only, in one study, overexpression of inhibitors of apoptosis proteins, including BIRC6, cIAP1, cIAP2, XIAP, Livin, and Survivin, was evaluated in 49 patients with ACC, and the results reported zero overexpression of BIRC6 in the studied cases [ 17 ]. The findings of this study diverge from those of the present study. Our observations indicate that the majority of ACC cases exhibited a higher percentage and intensity of BIRC6 expression, significantly surpassing that observed in normal salivary tissue. However, no notable difference was observed in the total expression level.

In addition, we found an increased percentage and intensity of BIRC6 expression in all three types of SGT, which confirms the results of previous studies in other cancer types . Dong *et al*. [ 9 ] evaluated BIRC6 expression in 78 samples with non-small-cell lung cancer (NSCLC) and showed that 52.6% of cases had moderate to strong BIRC6 expression and scored ≥1. Gharabaghi *et al*. [ 28 ] also showed high expression of BIRC6 in 75% of NSCLC tissue samples, significantly different from the weak expression in normal tissue cells. Li *et al*. [ 27 ] compared BIRC6 expression in 80 esophageal squamous cell carcinoma tissues with 80 paired normal tissues, and the results showed significantly higher levels of BIRC6 expression of RNA and protein in the carcinoma tissues. Others have also shown upregulation and overexpression of BIRC6 in oral lichen planus (with and without dysplasia), hyperkeratosis, oral squamous cell carcinoma, and oral epithelial dysplasia [ 29 ]. Studying the expression level of BIRC6 in epithelial ovarian cancer cells compared with normal tissue by Western blot showed a higher BIRC6 expression in the carcinoma tissue than in normal control tissue [ 10 ]. The evaluation of BIRC6 expression using IHC, Western blotting, and reverse transcription-quantitative polymerase chain reaction (PCR) in renal cancer tissues, compared with adjacent non-cancerous tissues and paired normal tissues, showed a higher expression in carcinoma tissues [ 30 ]. The results of these studies confirm those of the present study, indicating higher expression of BIRC6 in SGT cells compared with normal cells. However, further studies are required in this regard to approve BIRC6 as an important biomarker in SGTs. Furthermore, lower BIRC6 levels have been confirmed in some types of cancers, such as acute myeloid leukemia (AML). The study by Schläfli *et al*. [ 31 ] comparing AML cells with granulocytes from healthy donors, showed lower expression of BIRC6 in cancerous cells, which increased during neutrophil differentiation of AML cell lines. As the different behavior and progression of various cancer types can make a difference in the results of studies, evaluating BIRC6 expression in different cancers, it is necessary to evaluate each tumor type separately. 

Another important finding in the present study was the significantly higher percentage and intensity of BIRC6 expression in malignant types (ACC and MEC), compared with the benign type (PA), while the frequency of BIRC6 overexpression was not associated with patients’ sex or age, tumor size, and metastasis status. Previous studies on prostate cancer revealed an association between BIRC6 expression and more advanced stages of the disease. Elevated BIRC6 expression was noted at T3-4 stages compared to T1-2 stages in benign cases, along with cases exhibiting lymph node metastasis and prostatic capsule invasion [ 12 , 32 ]. Other studies have also shown a significant association of BIRC6 overexpression with tumor size and invasion depth in colorectal cancer [ 11 ] and childhood AML [ 31 ]. The results of other studies have shown more advanced pathological T stage, poor differentiation, and lymph node metastasis in cases with BIRC6 overexpression in NSCLC [ 28 , 33 ] and esophageal squamous cell carcinomas [ 27 , 29 , 34 ], compared with those with low expression of BIRC6. Renal cancer tissues with different expression levels also showed different T stage, nodal involvement, and tumor-node metastasis stage [ 30 ]. The results of the above-mentioned studies confirm the association of BIRC6 overexpression with tumor advancement in other cancer types [ 11 - 12 , 27 - 28 , 30 - 32 ]. Nevertheless, the results of the present study did not find any association with tumor size and metastasis status in SGT cells, although a significant difference was observed in higher percentage of BIRC6 expression between malignant and benign types. This may be because of the small sample size of the tissues investigated in each subtype of SGT. 

Furthermore, BIRC6 overexpression has been associated with worse overall survival and shorter disease-free survival in colorectal cancer [ 11 ], prostate cancer [ 32 , 35 ], and childhood AML and acute lymphoblastic leukemia (ALL) [ 31 , 36 ]. Other cancer types, such as NSCLC [ 9 ] and esophageal squamous cell carcinoma, have also been associated with shorter overall survival as well as lower relapse-free and disease-free survival rates [ 27 ]. However, we evaluated tissue samples and did not consider the patient’s prognosis or outcome.

This study was the first to evaluate BIRC6 expression in the three types of SGT, but had some limitations. Firstly, the retrospective nature of the study limited the evaluation of other clinical variables, response to treatment, and patient’s prognosis. Secondly, we only evaluated BIRC6 expression by IHC and did not use other methods, such as real-time PCR, Western blot, and enzyme-linked immunosorbent assay (ELISA). 

## Conclusion

The results of the present study showed a significant role for BIRC6 in SGT with a higher percentage and intensity of expression in malignant cancer types compared with the benign type and normal tissues. These preliminary results suggest BIRC6 as an appropriate biomarker for targeted anticancer therapy. However, further studies are required to determine the effect of BIRC6 on the prognosis and clinical outcome of patients with SGTs. 
